# Introducing the Proceedings of the CCP-EM Spring Symposium 

**DOI:** 10.1107/S2059798317008002

**Published:** 2017-06-01

**Authors:** Tom Burnley

**Affiliations:** a Scientific Computing Department, Science and Technology Facilities Council, Research Complex at Harwell, Didcot OX11 0FA, UK

**Keywords:** cryo-EM, CCP-EM Spring Symposium

## Abstract

An introduction to the CCP-EM proceedings special issue of *Acta Cryst D*.

This special issue is the inaugural edition of Proceedings of the CCP-EM Spring Symposium. The symposium is an annual conference which aims to review and highlight state-of-the-art developments in macromolecular cryo-electron microscopy in an accessible and convivial manner. To that end, we are privileged to follow in the footsteps of our successful sister project CCP4, and aim to replicate the popularity of the evergreen CCP4 Study Weekend. *Acta Crystallographica Section D* has published a collection of articles from that meeting since 1998, producing a compendium of insightful and often highly cited works. We hope that in 20 years’ time, the Spring Symposium proceedings may be viewed in a similar light.

Cryo-EM is currently in a golden age: multiple, symbiotic improvements in instrument technology have resulted in vastly improved signal-to-noise allowing a step-change in resolution and scale. Indeed such is the pace of development that the term ‘resolution revolution’, first coined in 2014, is fast becoming a cliché. This has propelled cryo-EM into the mainstream, evidenced by the exponential rise in the number of EM-derived structures deposited in the PDB and attracting high numbers of new researchers into the field. These new methods have opened a rich tranche of new biological systems to explore, including those that were well hidden from the bright light of other structural biology disciplines. As such, in this edition, we focus on emerging methods – the how, and highlight applications – the why.

The sharp increase in PDB models and experimental map depositions in the EMDB is quantified in the article from Ardan Patwardhan (EBI). Arising from that is the popularity of the *RELION* software package for single particle reconstruction (SPR). Its author is Sjors Scheres (MRC-LMB) and here he describes a new workflow for rapid, on-the-fly data processing in *RELION*. To fully exploit the exquisite high-resolution, phased maps produced by SPR, the volumes should be annotated *via* the fitting of atomic models. An article by myself, Colin Palmer and Martyn Winn introducing the *CCP-EM* software suite, connects multiple tools for the docking, refinement and validation of such structures. It should be noted that the ‘revolution’ is by no means limited to SPR only. The same hardware improvements produce evermore detailed tomograms and Daniel Castaño-Díez (University of Basel) describes the use of the *Dynamo* software package for subtomogram averaging in this issue. Of course these works hinge on the availability of experimental data and this, in turn, relies on the accessibility of high-end instrumentation. Daniel Clare (Diamond Light Source) and coworkers describe the first year of operation at the Electron Bio-Imaging Centre, the national UK facility.

The availability of high-resolution maps has shown the potential of using SPR as a tool for structure-based inhibitor design and sparked industrial interest in the technique and its application is expected to be important in the coming years. Within this issue, articles from Shaun Rawson (University of Leeds) and Paula da Fonseca (MRC-LMB) both provide reviews on this subject. Alessandro Costa (Crick Institute) reviews the application of cryo-EM to chromatin, highlighting where new techniques can provide more detail on important biological systems. Of course no structural biological technique should stand alone. The current generation of structural biologists are blessed with multiple instruments to dissect the inner workings of molecular machines, and each discipline has its own particular strengths. The work on the ribosome machinery presented by Abid Javed (UCL) is an illustration of such hybridization: utilising NMR in concert with EM. Furthermore, I would like to thank Abid for his own illustration as he kindly provided the cover art for this issue.

Thus far, CCP-EM has been funded primarily by the MRC so I would like to thank them for their support. I would also like to thank the authors for their respective contributions to this first edition along with thanking my co-editors Paula da Fonseca and Randy Read (University of Cambridge). The methods and applications presented are a glimpse of what can be expected in the coming years for cryo-EM. We hope to be able to present and document these exciting developments for many more years with the *Acta Crystallographica Section D* Proceedings of the CCP-EM Spring Symposium.

